# Fossil evidence for the ancient link between clonal fragmentation, six-fold symmetry and an epizoic lifestyle in asterozoan echinoderms

**DOI:** 10.1098/rspb.2023.2832

**Published:** 2024-05-15

**Authors:** Ben Thuy, Lea D. Numberger-Thuy, Jürgen Härer, Andreas Kroh, Viola Winkler, Günter Schweigert

**Affiliations:** ^1^ Department of Palaeontology, Natural History Museum Luxembourg, 25 rue Münster, 2160 Luxembourg; ^2^ Dinosaurierpark Teufelsschlucht, Ferschweilerstrasse 50, Ernzen, 54668 Germany; ^3^ Roennebergstraße 5, Berlin 12161, Germany; ^4^ Naturhistorisches Museum, Burgring 7 Vienna 1010, Austria; ^5^ Palaeontology Department, State Museum of Natural History Stuttgart, Rosenstein 1 Stuttgart 70191, Germany

**Keywords:** Ophiuroidea, late Jurassic, fissiparous, phylogeny, Ophiactidae

## Abstract

Asexual reproduction by means of splitting, also called fissiparity, is a common feature in some asterozoan groups, especially in ophiactid brittle stars. Most fissiparous brittle stars show six instead of the usual five rays, live as epibionts on host organisms, and use clonal fragmentation to rapidly colonize secluded habitats and effectively expand the margins of their distribution area. While the biology and ecology of clonal fragmentation are comparatively well understood, virtually nothing is known about the evolution and geological history of that phenomenon. Here, we describe an exceptional fossil of an articulated six-armed brittle star from the Late Jurassic of Germany, showing one body half in the process of regeneration, and assign it to the new species *Ophiactis hex* sp. nov. Phylogenetic inference shows that the fossil represents the oldest member of the extant family Ophiactidae. Because the *Ophiactis hex* specimen shows an original six-fold symmetry combined with a morphology typically found in epizoic ophiuroids, in line with recent fissiparous ophiactid relatives, we assume that the regenerating body half is an indication for fissiparity. *Ophiactis hex* thus shows that fissiparity was established as a means of asexual reproduction in asterozoan echinoderms by the Late Jurassic.

## 1. Introduction

Fragmentation is a form of asexual reproduction where a multicellular organism is split into fragments, each of which develops into fully grown individuals that represent clones of the original organism [1,2]. Whereas corals and sponges commonly split as a means of proliferation, clonal fragmentation is rarely seen in more complex animals, in part because of the extraordinary regeneration potential required to ascertain the viability of the organismal fragments. One of the few highly organized metazoan clades that routinely resort to clonal fragmentation as a mode of postlarval asexual reproduction are the asterozoan echinoderms (i.e. sea stars and brittle stars) [3,4]. The most common form of fragmentation in these animals is a separation along a median fission plane, splitting the original organism into two halves that each regrow the missing parts, a process called fissiparity.

In recent asterozoans, some 32 species of ophiuroid and 17 species of asteroid (table 1) are known to be capable of reproducing asexually by fission [3,5,6]. Although these species are distributed over a range of phylogenetically distant family level clades, they share a number of striking anatomical and behavioural similarities. Fissiparous species, or more precisely the fissiparous phenotype of these species, tend to have a six-fold symmetry rather than the typical five-fold symmetry that characterizes the echinoderm body plan, probably to ease division into two subequal halves [3]. Furthermore, they tend to have a small body size, thus achieving quick regeneration after fission [7]. Finally, at least in brittle stars, fissiparous species are often epizoic, living on a host organism such as a sponge, a macroalga or a cnidarian [8]. It has been assumed previously that the asexual reproduction mode allowed epizoic asterozoan species to rapidly colonize small, secluded habitats such as algal tufts and sponge aggregations [9] and to effectively expand the margins of their distribution area [10]. It is, however, no coincidence that the most rapidly spreading invasive asterozoan species in the world oceans are fissiparous [11].

**Table 1.** List of all currently known fissiparous asteroid and ophiuroid species [5,6].

superorder	family	species
Class Asteroidea		
Valvatacea	Asterinidae	*Aquilonastra anomala* (H.L. Clark, 1921)
		*Aquilonastra burtoni* (Gray, 1840)
		*Ailsastra heteractis* (H. L. Clark, 1938)
		*Nepanthia belcheri* (Perrier, 1875)
		*Pseudonepanthia briareus* (Bell, 1894)
Forcipulatacea	Asteriidae	*Coscinasterias acutispina* (Stimpson, 1862)
		*Coscinasterias calamaria* (Gray, 1840)
		*Coscinasterias tenuispina* (Lamarck, 1816)
		*Sclerasterias alexandri* (Ludwig, 1905)
		*Sclerasterias euplecta* (Fisher, 1906)
		*Sclerasterias heteropaes* Fisher, 1924
		*Sclerasterias richardi* (Perrier in Milne-Edwards, 1882)
		*Stephanasterias albula* (Stimpson, 1853)
	Stichasteridae	*Allostichaster capensis* (Perrier, 1875)
		*Allostichaster insignis* (Farquhar, 1895)
		*Allostichaster palmula* Benavides-Serrato & O'Loughlin, 2007
		*Allostichaster polyplax* (Müller & Troschel, 1844)
Class Ophiuroidea		
Euryophiurida	Euryalidae	*Astroceras annulatum* Mortensen, 1933
		*Astrocharis ijimai* Matsumoto, 1911
		*Astrocharis virgo* Koehler, 1904
		*Asteromorpha koehleri* (Döderlein, 1898)
		*Asteromorpha rousseaui* (Michelin, 1862)
		*Asteromorpha tenax* Baker, 1980
		*Asteroschema bidwillae* McKnight, 2000
		*Asteroschema wrighti* McKnight, 2000
Ophintegrida	Ophiomyxidae	*Ophiostiba hidekii* Matsumoto, 1915
	Ophiacanthidae	*Ophiacantha scissionis* Lee, Stöhr, Bae & Shin, 2019
	Ophiocomidae	*Ophiocomella ophiactoides* (H.L. Clark, 1900)
		*Ophiocomella sexradia* (Duncan, 1887)
	Ophioleucidae	*Ophiostriatus sexradiatus* Irimura, 1993
	Ophionereididae	*Ophionereis dubia* (Müller & Troschel, 1842)
		*Ophionereis sexradia* Mortensen, 1936
	Hemieuryalidae	*Astrogymnotes catasticta* H.L. Clark, 1914
		*Ophioholcus sexradiata* (Koehler, 1914)
	Amphiuridae	*Amphiura (Amphiura) velox* Koehler, 1910
		*Amphipholis torelli* Ljungman, 1872
		*Amphiodia dividua* Mortensen, 1933
		*Amphioplus (Amphioplus) hexabrachiatus* Stöhr, 2003
	Ophiactidae	*Ophiactis macrolepidota* Marktanner-Turneretscher, 1887
		*Ophiactis modesta* Brock, 1888
		*Ophiactis muelleri* Lütken, 1856
		*Ophiactis nidarosiensis* Mortensen, 1920
		*Ophiactis plana* Lyman, 1869
		*Ophiactis profundi* Lütken & Mortensen, 1899
		*Ophiactis savignyi* (Müller & Troschel, 1842)
		*Ophiactis seminuda* Mortensen, 1936
		*Ophiactis simplex* (LeConte, 1851)
		*Ophiactis virens* (M. Sars, 1859)
	Ophiotrichidae	*Ophiothela mirabilis* (Verrill, 1867)

While the biology and physiology of clonal fragmentation in echinoderms is comparatively well-studied, virtually nothing is known about the evolutionary history of this phenomenon. The phylogenetic distribution across largely unrelated clades [3] suggests recurrent evolution of fissiparity in asterozoans, but in the absence of fossil evidence, no conclusions can be drawn as to when and where in Earth history this phenomenon evolved for the first time. Fossil evidence for fissiparity is only visible in asterozoan skeletons that are preserved as articulated specimens, frozen in time while regenerating the missing body half. Given the rapid post-mortem decay of the multi-element asterozoan skeleton, generally precluding fossilization of intact individuals, and given the limited time window during which the regenerating half is visible as such, fissiparity is highly unlikely to be preserved in the fossil record.

Here, we describe an exceptional ophiuroid fossil from the Jurassic of Germany showing one body half in the process of regeneration. We study the phylogenetic relationships of the new ophiuroid to resolve its position within the evolutionary history of the Ophiuroidea. Based on key anatomical features of the specimen and its geological context, we investigate the palaeobiology of the new ophiuroid from the perspective of fissiparity.

## 2. Material and methods

The holotype (and only known specimen) of the new species was collected from the Upper Jurassic Nusplingen Lithographic Limestone. This rock unit corresponds to the Nusplingen Formation and represents a 10–15 m thick series of marine laminated limestones with intercalated turbidites and bioturbated limestones [12–14]. The site is located in the south-western part of the Swabian Alb in southern Germany, 12 km north of the river Danube valley, west of the village of Nusplingen. The Nusplingen Formation is well-known for its exceptionally preserved fossils (e.g. pterosaurs, thalattosuchian crocodiles, sharks, decapod crustaceans). The age of the Nusplingen Formation is dated into the late Kimmeridgian Beckeri Zone (Ulmense Subzone) by its characteristic ammonite fauna [15].

Based on field data, lithofacies analysis, taphonomic observations and isotopic studies on belemnite rostra and shark teeth [16–18], the Nusplingen Lithographic Limestone is reconstructed as a deep lagoonal setting with an estimated palaeodepth of 80–100 m, bordered by steep margins and surrounded by siliceous sponge-microbial mounds, subordinate coral meadows, shallow areas with calcareous arenites and oolites and shallow pelagic islands [16,19]. Only the deepest parts of the lagoonal deposits are accessible, whereas coeval sediments in the surroundings were completely eroded in post-Jurassic times. The lamination of the Nusplingen Lithographic Limestone results from oxygen depletion at the seafloor that hampered benthic life and bioturbation. In the intercalated turbidite beds and mudflows an allochthonous fauna of siliceous sponges, brachiopods, echinoids, bivalves and crabs occurs, typical of sponge-microbial mounds surrounding the lagoon. Siliceous sponges furthermore occur within the laminated limestones where their spicules form a major component of the sediment [12,13,20,21]. The sponges are especially abundant in beds consisting of finely splitting laminates; this is exactly the same lithology that has delivered the brittle stars specimen described herein.

The ophiuroid specimen described in the present paper was discovered during the 2018 excavation campaign by the State Museum of Natural History Stuttgartin the 20-cm-thick, finely laminated bed ‘M' of the section. The bed in question yielded sponges, pelagic crinoids (*Saccocoma*), belemnite rostra, coprolites of ammonites (*Lumbricaria*), and ammonite beaks (aptychi). The exact finding horizon of the ophiuroid lacks any evidence of bioturbation. Preparation was carried out mechanically under a binocular equipped with ultraviolet illumination using various needles. The specimen is part of the palaeontological collection of the Stuttgart Natural History Museum (SMNS). Light microscope images were taken at the National Museum of Natural History in Luxembourg using a Keyence VHX-6000 digital microscope. To visualize the skeletal features inside the disc and on the ventral side of the individual, the specimen was imaged using X-ray microcomputed tomography (microCT) using an YXLON FF35CT system at the Natural History Museum in Vienna (NHMW). The equipped FXE transmission beam was used with a tube voltage of 45 kV and current of 110 µA. A total of 3600 projection images were taken with an exposure time of 2 s each. The reconstructed dataset, with an isotropic voxel size of 7.1 µm, was visualized using the Dragonfly software, Version 2022.1 for Windows (Object Research Systems, Montreal, Canada, 2022).

We use the morphological terminology by Stöhr *et al*. [22], Thuy & Stöhr [23,24] and Hendler [25], and adopt the classification by O'Hara *et al*. [26,27]. For the purpose of the phylogenetic estimate, we scored the skeletal features following Thuy & Stöhr [24,28], based on the same character definitions and acronyms and using the character matrix elaborated by Thuy & Stöhr [24] and modified by Thuy & Stöhr [28] and Thuy *et al*. [29]. The character matrix including the scores of the ophiuroid described in the present paper is part of the electronic supplementary material, information. Since the goal of the Bayesian inference analysis was to assess the family level position of the new fossil rather than its relationships within the Ophiactidae, we refrained from including additional recent *Ophiactis* species. Bayesian inference analysis was performed using MrBayes [30] using a modified version of the Juke-Cantor model for morphological data as outlined by Lewis [31], with variable character states from 2 to 10 [32]. Only variable characters were sampled. Character selection bias was compensated for by letting MrBayes search for parsimony informative characters (Mkpars model) [32]. All character states were treated as having equal frequency, and prior probabilities were equal for all trees. We assumed that evolutionary rates vary between sites according to a discrete gamma distribution. Average standard deviations of split frequencies stabilized at about 0.007 after 3 million generations (mgen), sampled every 1,000 generations. The first 25% of the trees were discarded as burnin. The consensus trees were examined with the software FigTree v. 1.4.2 by Rambaut (http://tree.bio.ed.ac.uk/software/figtree/). Confidence intervals of 95–99% were considered as strong support for a node to be true, and at least 90% probability as good support.

## 3. Nomenclatural acts

This published work and the nomenclatural acts it contains have been registered in ZooBank, the proposed online registration system for the International Code of Zoological Nomenclature (ICZN). The ZooBank LSID (Life Science Identifiers) for this publication is urn:lsid:zoobank.org:pub:94C95B38-7C93-4626-AC64-CE5E5727B947. The LSID for the new taxa are given below.

## 4. Systematic palaeontology

Class Ophiuroidea Gray, 1840

Superorder Ophintegrida O'Hara *et al*., 2017

Order Amphilepidida O'Hara *et al*., 2017

Superfamily Ophiactoidea Ljungman, 1867

Family Ophiactidae Matsumoto, 1915

Genus *Ophiactis* Lütken, 1856

*Ophiactis hex* sp. nov.

LSID: urn:lsid:zoobank.org:act:B4C66E07-9B00-4041-BD3A-322714A8FCFC

### (a) Holotype and type locality data

SMNS 70508; Nusplingen Quarry on top of Westerberg hill, Nusplingen, Germany; Nusplingen Lithographic Limestone (Nusplingen Formation), Beckeri Zone, Ulmense Subzone, late Kimmeridgian, Late Jurassic.

### (b) Etymology

Species name (used as noun in apposition) referring to Hex, the organic/inorganic/magical super-computer of Terry Pratchett's Unseen University, capable of thinking the unthinkable.

### (c) Species diagnosis

Small ophiactid with six arms, dorsal side of disc with a dense cover of granules and spinelets; lateral arm plates with outer surface covered by small tubercles arranged in a faint vertical striation; arm spines large and slightly flattened; distalmost arm segments with at least one hook-shaped arm spine.

### (d) Holotype description

**Figure 1.**
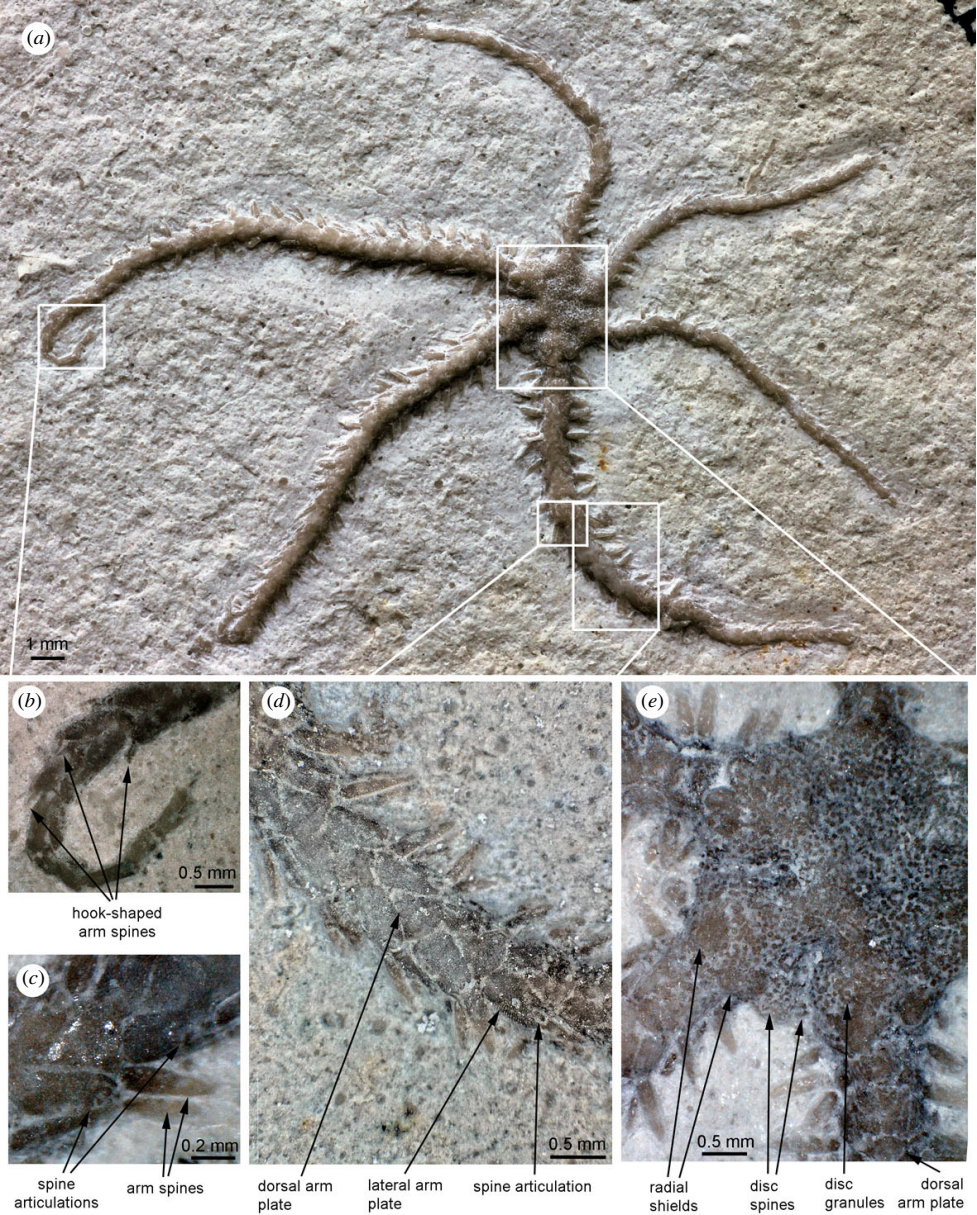
*Ophiactis hex* sp. nov., holotype SMNS 70508; from the Nusplingen Lithographic Limestone (Nusplingen Formation), Beckeri Zone, Ulmense Subzone, late Kimmeridgian, Late Jurassic, Nusplingen, Germany. Light photographs of the complete specimen (*a*) exposing the dorsal side, with details of an arm tip (*b*), median arm segments (*c,d*), and the disc (*e*). Rectangles in (*a*) show positions of close-ups (*b*) to (*e*).

**Figure 2.**
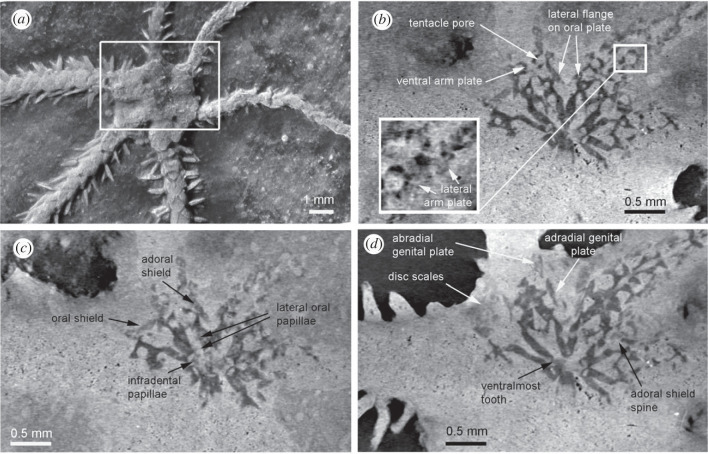
*Ophiactis hex* sp. nov., SMNS 70508; from the Nusplingen Lithographic Limestone (Nusplingen Formation), Beckeri Zone, Ulmense Subzone, late Kimmeridgian, Late Jurassic, Nusplingen, Germany. MicroCT scans showing the surface (*a*) of the specimen and sections at various depths (*b–d*) including a close-up of proximal lateral arm plates in (*b*). Rectangle in (*a*) shows position of (*b*) to (*d*).

Small, fully articulated ophiuroid (figure 1*a*) on a limestone slab, exposing the dorsal side; disc diameter 3.4 mm, length of longest arm 15.5 mm; body divided in two unequally sized halves; disc radius (arm base to disc centre) measured at three smaller rays (1.6 mm, 1.6 mm and 1.7 mm, respectively) and three larger rays (2.0 mm, 2.0 mm and 2.1 mm, respectively) showing significant disc size difference; disc round to slightly hexagonal; spherical granules (figure 1*e*) densely covering the small, thin disc scales, intermingled with short, conical disc spines in interradii; radial shields (figure 1*e*) small, equalling three fifths of the disc radius in length, with rounded distal-adradial portion exposed and proximal portion covered by disc granules, pairs of radial shields in contact distally; oral skeleton made visible using microCT scanning (figure 2), showing ventral interradii covered by thin scales (figure 2*d*), presence of granules and/or spines on interradial disc scales not discernible; large, slender oral plates with a well-developed flange (figure 2*b*), forming long jaws; large, cordiform ventralmost tooth (figure 2*d*); small, blunt, spine-like infradental papillae on lateral edge of dental plate, two small, elongate, blunt lateral oral papillae and a single large adoral shield spine per half jaw (figure 2*c*); relatively short, slender adoral shields (figure 2*c*); oral shields partly discernible, showing obtuse proximal angle with straight edges (figure 2*c*); abradial genital plate short, relatively wide, with a concave distal tip (figure 2*d*); adradial genital plate almost two times longer than adradial one. Six arms, relatively short, two of the three larger arms with a regenerating tip (figure 1*a,b*); dorsal arm plates large, fan-shaped, as long as wide in proximalmost segments, slightly elongate in all others, with a finely tuberculate surface (figure 1*d*); lateral arm plates (figure 1*c*) abutting dorsally in most arm segments except for the proximalmost; outer surface covered by small tubercles aligned into a fine vertical striation; spine articulations large (figure 1*c*), freestanding on distal edge of lateral arm plates, composed of two separated lobes, dorsal one bent and larger than the ventral one; at least three arm spines (figure 1*d*), large, laterally slightly flattened, with tiny lateral thorns, as long as one arm segment; dorsalmost arm spine longest; distalmost arm segments with at least one small hook-shaped spine (figure 1*b*); inner side of lateral arm plates unknown; ventral portion of lateral arm plates (figure 2*b*) protruding ventro-proximalwards, encompassing a large tentacle opening; ventral arm plates (figure 2*b*) slightly wider than long in proximal arm segments, with deep tentacle notch.

### (e) Taxonomic affinities

The ophiuroid specimen described in the present paper shows a combination of morphological features that is exclusively found in the extant family Ophiactidae within the order Amphilepidida. These consist of freestanding spine articulations composed of a ventral lobe fully separated from a larger, bent dorsal lobe, lateral arm plates with a finely tuberculate outer surface, infradental papillae on the lateral edge of the dental plate, and oral plates with a well-developed flange [27]. The family Ophiactidae, as currently defined, comprises the type genus *Ophiactis*, a morphologically diverse complex of more than 50 extant species, and the genus *Hemipholis* with only two extant species [27]. The presence of disc scales in the ventral interradii in the ophiuroid specimen described herein rules out *Hemipholis* [33]. Assignment to *Ophiactis* is supported by the majority of the morphological characters, except for the dense cover of granules on the dorsal disc. Given the morphological heterogeneity of *Ophiactis*, however, we refrain from introducing a new genus and assign our new fossil to *Ophiactis*, pending a revision and possible subdivision of the genus.

The fossil *Ophiactis* described in the present paper differs from all recent congeners and from the sole unambiguous fossil species, *Ophiactis applegatei* Martin-Medrano, Thuy & Garcia-Barrera, 2009 from the Early Cretaceous of Mexico [34], in the dense cover of granules and spinelets on the dorsal disc. The fossil lateral arm plates from the latest Cretaceous of Germany [35] described as *Ophiactis*? *sulcata* Kutscher & Jagt, 2000 show a combination of characters that precludes assignment to *Ophiactis* or the Ophiactidae in general. Instead, the spine articulation morphology and the shape of the vertebral articular structure suggests a basal position within the Ophionereididae. The holotype specimen of *Ophiothrix*? *cristata* Kutscher & Jagt, 2000, also from the latest Cretaceous of Germany [35], in contrast, shows a typical ophiactid morphology. We therefore suggest transferring that species to *Ophiactis*, thus proposing the new combination *Ophiactis cristata* (Kutscher & Jagt, 2000). It differs from the species described herein by having very coarse outer surface tubercles not merged into a vertical striation. Because the specimen described in the present paper differs from all other fossil and recent ophiactids, we describe it as a new species. It represents the oldest representative of the Ophiactidae known to date and pushes the origin of the family back to the Jurassic.

### (f) Phylogeny

**Figure 3.**
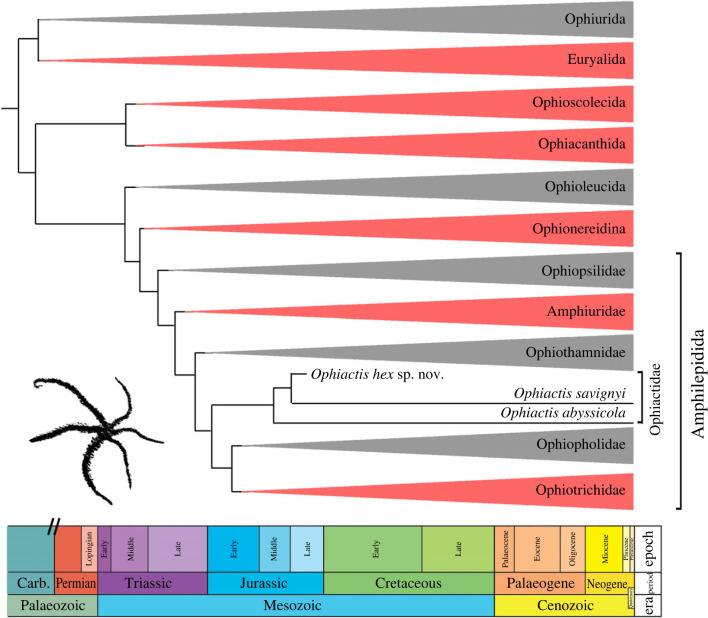
Evolutionary tree inferred from Bayesian inference analysis (see electronic supplementary material) and previously published phylogenomic trees [26,36] to show the position of *Ophiactis hex* sp. nov. Clades in red include at least one fissiparous species.

In order to determine the phylogenetic position of the new ophiuroid, we performed a morphological cladistic analysis using Bayesian inference (electronic supplementary material, information). We integrated the six-armed specimen described in the present paper in the matrix used by previous studies on post-Palaeozoic ophiuroids, comprising representatives of all major extant clades and a number of key fossil taxa. The resulting tree (electronic supplementary material, information; figure 3) indicates a position of the new fossil within the clade formed by the two sampled recent *Ophiactis* species and thereby confirms assignment to the Ophiactidae. The Late Jurassic age and phylogenetic position of *Ophiactis hex* furthermore sets a new minimum age for the ophiactid node and thereby implies that the more basal amphilepidid clades diverged much earlier than suggested previously [36]. While the greater age for the ophiactid and the more stemward amphilepidid nodes challenges previous molecular clock estimates [36], it aligns with recent studies showing the early crown-group diversification took place much earlier than previously assumed [37,38].

## 5. Discussion

While skeletons of ophiuroids with individual arms frozen in the process of regeneration are relatively common in the fossil record, cases of individuals with a regenerating body half are exceedingly rare. To the best of our knowledge, the specimen described in the present paper is only the second case known so far, and the first one for which regeneration seems indeed linked to six-fold symmetry and clonal fragmentation. The other case of a fossil ophiuroid with a regenerating body half is a six-armed individual of the ophiomusin *Enakomusium gagnebini* (Thurman, 1851) from the Oxfordian, Upper Jurassic, of Switzerland [39]. The latter species shows the typical morphology of an epizoic bottom dweller, i.e. a relatively large body size, stout overall plating, relatively stiff, cylindrical arms and tiny, adpressed arm spines, and is known from dozens of articulated specimens to be five-armed [39]. In addition, the three non-regenerating arms of the *E. gagnebini* specimen show an interbrachial angle of approximately 70°, compared to 60° in *Ophiactis hex*. This suggests that the six-fold symmetry in the regenerating *E. gagnebini* specimen is most probably the result of a sublethally injured, originally pentamerous animal rather than a fissiparous clone.

By contrast, the *Ophiactis hex* specimen described in the present paper shows a regular six-fold symmetry without any signs of accidental ray addition, suggesting that the individual was hexamerous before fragmentation. With only one specimen at hand, however, it is impossible to conclude whether the species was regularly six-armed or not. What can be generalized to the species level, however, is the assumption of an epizoic lifestyle. *Ophiactis hex* shows a number of features that are typically found in ophiuroids living epizoic on hosts [40], in particular (1) a relatively small body size, (2) comparatively short, flexible arms, (3) stout, erect arm spines on proximal to median arm segments, and (4) hook-shaped arm spines on distal arm segments. Potential host organisms could be the siliceous sponges that grew abundantly in the areas surrounding the lagoon, as evidenced by the numerous sponge spicules and common sponge fragments in the rocks yielding the ophiuroid specimen.

The vast majority of the recent fissiparous ophiuroid species have a regular six-fold symmetry and an epizoic life style [2,3]. This strongly suggests that the regenerating body half of the unique *Ophiactis hex* specimen is indeed indicative of clonal fragmentation. The family-level position of the species lends further support to the hypothesis of clonal fragmentation. In fact, the Ophiactidae hold by far the highest share of fissiparous species. A total of 10 out of 52 currently accepted ophiactid species, i.e. almost 20% of the family's recent diversity [5], are known to reproduce by splitting. Thus, *Ophiactis hex* seems to represent the first fossil case of a fissiparous ophiuroid. It suggests that clonal fragmentation, combined with six-fold symmetry and an epizoic lifestyle, was established as a means of asexual reproduction in ophiuroids by the Late Jurassic.

## Data Availability

The data are provided in electronic supplementary material [41].
